# Noncontact 3D evaluation of surface topography of reciprocating instruments after retreatment procedures

**DOI:** 10.1590/0103-6440202204876

**Published:** 2022-06-24

**Authors:** Miriam Fatima Zaccaro-Scelza, Renato Lenoir Cardoso Henrique Martinez, Sandro Oliveira Tavares, Fabiano Palmeira Gonçalves, Marcelo Montagnana, Emmanuel João Nogueira Leal da Silva, Pantaleo Scelza

**Affiliations:** 1 Department of Endodontics, Faculty of Dentistry, Universidade Federal Fluminense, Niterói, RJ, Brazil.; 2 Post-Graduate Program in Dentistry, Universidade Federal Fluminense, Niterói, RJ, Brazil; 3 Taylor Hobson of Brazil, São Paulo, SP, Brazil.; 4 Geriatric Dentistry Department, Faculty of Dentistry, Universidade Federal Fluminense, Niterói, RJ, Brazil.

**Keywords:** Imaging Three-dimensional, Endodontic retreatment, Dental materials

## Abstract

This study evaluated the Reciproc R25 and Pro-R 25 instruments in unused condition, after one and a second use in endodontic retreatment employing a noncontact 3D light interferometer profiler, scanning electron microscopy (SEM) and cyclic fatigue tests. Twenty single-root teeth were instrumented with Reciproc R25 and filled with gutta-percha and sealer. A 3D profiler with a 20x objective using the Mx™ software was used to evaluate the cutting blade surfaces of Reciproc R25 and Pro-R 25 (n=5 per group) in unused condition, after the first and second uses in retreatment procedures. After retreatment, SEM was used to evaluate the topographic features of the used instruments. Cyclic fatigue tests were performed to compare new to used instruments. One-way ANOVA followed by Tukey test was used to compare the tested instruments before and after the first and second uses. Student t-test was used to compare the different instruments and for cyclic fatigue evaluation. No significant differences were observed in the cutting blade surfaces of Reciproc and Pro-R before and after one and two uses (p>0.05). Reciproc without use showed higher Sa and Sq when compared to Pro-R without use (p<0.05). No differences were observed between Reciproc and Pro-R after one and two uses (p>0.05). New and unused Reciproc showed longer time to fracture than Pro-R instruments (p<0.05), and only Pro-R showed differences between new and used instruments (p<0.05). Retreatment procedures with Reciproc and Pro-R did not change the surface topography of instruments. Reciproc had greater resistance to cyclic fatigue compared with Pro-R.

## Resumo

Este estudo avaliou os instrumentos Reciproc R25 e Pro-R 25 sem uso, após um primeiro e um segundo uso em retratamento endodôntico com perfilômetro 3D por interferometria de luz sem contato, microscópio eletrônico de varredura (MEV) e testes de fadiga cíclica. Vinte dentes unirradiculares foram instrumentados com Reciproc R25 e obturados com guta-percha e cimento endodôntico. Um perfilômetro 3D com uma objetiva 20x usando o software Mx ™ foi usado para avaliar as superfícies da lâmina de corte do Reciproc e Pro-R (n = 5 por grupo) na condição sem uso, após o primeiro e após um segundo uso em procedimentos de retratamento. Após retratamento, o MEV foi usado para avaliar as características topográficas dos instrumentos utilizados. Testes de fadiga cíclica foram realizados para comparar instrumentos novos com instrumentos usados. O teste One-way ANOVA seguido pelo teste de Tukey foi usado para comparar os instrumentos testados nos diferentes estágios. Para comparar os diferentes instrumentos e para avaliação de fadiga cílcica, foi utilizado o Student t-test. Não foram observadas diferenças estatisticamente significantes nas superfícies das lâminas de corte dos instrumentos Reciproc e Pro-R antes e após um e dois usos (p> 0,05). O Reciproc na condição sem uso apresentou maior Sa e Sq quando comparado ao Pro-R (p <0,05). Não foram observadas diferenças entre Reciproc e Pro-R após um e dois usos (p> 0,05). O Reciproc sem uso apresentou maior tempo de fratura do que os instrumentos Pro-R (p<0,05), e apenas o Pro-R apresentou diferenças entre instrumentos novos e usados (p<0,05). Os procedimentos de retratamento com Reciproc e Pro-R não alteraram a topografia da superfície dos instrumentos. Reciproc apresentou maior resistência à fadiga cíclica em comparação com o Pro-R.

## Introduction

Non-surgical root canal retreatment is still considered to be the first option when there is failure in the root canal treatment, mainly because it is a more conservative option ^1^ ^,^ ^2^ ^,^ ^3^. The evolution of techniques and instruments for root canal retreatment associated with a better understanding of the failures involved, have a direct influence on its success rate ^4^. The preparation of the root canal can result in wear and deformation of nickel-titanium (NiTi) instruments ^5^ as well as their unforeseen failure within root canals ^6^. Thus, there is a concern that these instruments may fracture within their elastic limit, with or without visible signs of previous deformation ^6^ ^,^ ^7^.

There are several instruments and techniques used to remove the filling material of root canals ^8^ and it is now well-known that the reciprocating kinematics made the procedure faster and more efficient ^4^. Moreover, the literature points out that reciprocating kinematics have the advantage, of a low incidence of fracture and deformations ^9^ ^,^ ^10^ ^,^ ^11^.

The manufacturing process of NiTi instruments can cause surface defects, such as deformations, debris, grooves, cracks, steps and microcavities. This can create stress in areas associated with initial cracks and propagate them, accelerating their fatigue and making the failure mechanism of material irreversible ^12^ ^,^ ^13^. The three-dimensional noncontact light interferometer profiler (3D profiler) allows for the qualitative and quantitative analysis of instruments before and after their use, providing a precise reproduction of topographic surface. Therefore, they are considered an accurate method for evaluating the surface of NiTi instruments ^14^ ^,^ ^15^. While most of the current available NiTi instruments are indicated for single use, the analysis of such instruments in more than one cycle is justified as the same instrument are used for the preparation of all the tooth canals, as in molars with 3 or 4 root canals.

Considering the relevance of surface defects on NiTi endodontic instruments, and the possibility of failures resulting from these defects that can be present in new instruments but also be generated after retreatment procedures, the present study quantitatively evaluated, using a 3D profiler, the surface topography of two different types of NiTi reciprocating instruments, Reciproc 25 and .08v taper (VDW, Munich, Germany) and Pro-R Retreatment 25 and .08v taper (MK Life, Porto Alegre, RS, Brazil) in unused condition, after one and a two uses in endodontic retreatment in human single-rooted teeth. As complementary analysis, scanning electron microscopy (SEM) of the used instruments and cyclic fatigue tests comparing new and used instruments were also performed.

## Materials and methods

### Root canal preparation

After approval by the local ethics committee (CAAE 23639019.0.0000.5243), twenty human single-root mandibular permanent canines, which were recently extracted for periodontal reasons. Teeth were selected using the following inclusion criteria: straight root canals, closed apices, intact root structure, and without previous root canal treatment, that allowed the adjusting of #15 K file in the root canal, evidenced by tactile perception and radiographic assessment. Teeth in which the #15 K file did not fit into the root canal were excluded. The crown was removed in order to obtain root segments of standard size in 10 mm. The working length established by insertion of a size # 10 K file (Dentsply-Sirona, Baillagues, Switzerland) placed up to the apical foramen and visualized under magnification. The root canals were instrumented with Reciproc R25 (VDW, Munich, Germany) under irrigation of 10 mL of 2.5% sodium hypochlorite (NaOCl) (Fórmula and Ação, São Paulo, SP, Brazil) and smear-layer removal was performed with 10 mL of 10% citric acid (Fórmula and Ação, São Paulo, SP, Brazil). The root canals were filled with Reciproc R25 gutta-percha cone (VDW) and AH Plus sealer (Dentsply-Sirona) using single-cone technique. The teeth were stored in 100% humidity at 37 ºC for 30 days before retreatment procedures. Then, teeth were randomly allocated using a coin toss between the Reciproc and Pro-R groups.

### Root canal retreatment

A sample size calculation was performed on GPower v3.1.3 software (University of Düsseldorf; Düsseldorf, Germany) based on the study of AlRahabi & Atta ^16^ with an effect size of 1.95, alpha-type error level of 0.05, a beta power of 0.8, resulting in five samples (instruments) per group. Thus, five unused Reciproc R25 (lot no. 229298 - VDW) and five unused Pro-R size 25 (lot no. 20180713 - MK Life) were used in the retreatment procedures. Each instrument was used on two different teeth.

The root canal retreatment procedures were standardized. The removal was carried out only mechanically, with the exclusive use of reciprocating instruments; there was no use of solvents. A single operator performed the procedures and both Reciproc and Pro-R were activated by means of a 6:1 reducing contra-angle headpiece (Sirona Dental Systems GmbH, Bensheim, Germany) coupled to a Silver Reciproc engine (VDW) in Reciproc ALL mode, according to the guidelines of instrument manufacturers.

From the beginning to the end of the procedure, the irrigation solution used was 2.5% NaOCl, always in the volume of 2.5 ml. The solution was maintained in the root canal while using the instrument. An in-and-out peck motion was performed in the apical direction with an amplitude of 3 mm. After each use, the instrument was cleaned with gauze that had been moistened with irrigation solution. This protocol was repeated until the entire length of the root canal had been reached. After reaching the full working length, lateral brushing motion was performed to prepare around the entire canal circumference. Instruments were used until no gutta-percha residues were observed neither on the instrument nor within the root canal. The removal of the filling material was verified by direct visualization and through teeth radiography. After this step, the instrument was washed in an ultrasonic vat for five minutes, dried with gauze and taken for surface evaluation in a three-dimensional (3D) noncontact light interferometer profiler. Then, the same instrument was used again in another root canal, respecting the procedure described previously.

### NiTi Instrument analysis in Light Interferometry Profiler

The sample analytical procedures were standardized in order to guarantee the reproducibility and accuracy of the measurements of the same cutting blade area on the surface of the instruments at different times, according to the method proposed by Ferreira et al. ^15^ and Ferreira et al. ^17^. Instruments were evaluated qualitative and quantitatively in unused condition, after a first and a second retreatment procedure. The measurement and analytical processes were performed using a Light Interferometry Profiler model NewView 8000 (Zygo Corporation, Middlefield, CT, USA) surface noncontact profilometer with a 20x objective lens, and the Mx™ Software (Zygo Corp).

First, a point on the instrument was chosen to serve as a rotational reference, which means the flatter area of the instrument's axis when it is inserted in the contra-angle. This allows direct visualization of the opposite face of the helix in a 180° rotation. Then, getting a reference 0 to the depth is easily reproduced for future measurements of *x*. The same marking process was performed on the opposite face of the helix. The instrument cable was then fixed to a support that was attached to the base of the motorized *x*/*y* table. The measurement areas were then defined by first positioning the equipment’s autofocus lens over the marking, point 0. The marking image displayed in the center of the computer screen will then serve to record numerical values ​​for the *x*, *y,* and *z* coordinates for each individual sample, to allow repeatable positioning, maximizing the accuracy for re-measuring the same position in the future.

Measurements and analyses were performed on two opposite surfaces (A - rotated 180° - B) of 166µm X 166µm, 3mm away from the tip of each instrument (measurement point). Applying the instrument's rotational reference to reproduce the same measurement in future tests, based on the distance of 350 µm, on the flank, from the crest of the cutting blade. From the reading of point 0, the motorized table was moved across until reaching the tip of each instrument and, subsequently, from the tip to the measurement point.

The numerical values of the *x*, *y* and *z* coordinates corresponding to the measurement areas were analyzed using four quantitative parameters of amplitude:

- Sa (average between the deviations of the peaks and valleys from a surface) represents the arithmetic mean of the height of the peaks and the depth of the valleys in relation to the average plane of the 3D measured area
- Sq (the root mean square roughness) - represents the height distribution in relation to the medium plane of the 3D measured area
- Sz - Describes the height of the maximum peak to the maximum valley in all the analyzed 3D area (ISO 25178-2)
- Ssk - Asymmetries in the distribution of peaks and valleys. The asymmetry parameters evaluate the relative position of the surface in relation to the mid plane

### Scanning electron microscopy (SEM)

After retreatment procedures and light interferometry profiler evaluation, a scanning electron microscope (SEM; JSM 5800; JEOL, Tokyo, Japan) was used to evaluate the topographic features of the instruments at 100 and ×250 magnifications.

### Cyclic fatigue

New instruments and instruments used after the retreatment procedures was tested regarding their cyclic fatigue (n=5). For this, new and second used Pro R and Reciproc instruments were mounted on a 6:1 reduction handpiece (VDW/Sirona Dental Systems, Bensheim, Germany) powered by a Silver Reciproc motor (VDW GmbH, Munich, Germany) and coupled on a tube model custom-made device (Odeme Dental Research, Luzerna, Santa Catarina, Brazil). The tests were conducted on a 6 mm radius and 86 degrees of curvature artificial canal having glycerin as a lubricant in a reciprocation kinematic using the program RECIPROC ALL, at room temperature (20ºC), which is in accordance to ASTM NiTi superelastic materials tensile testing international guidelines ^18^. The files were activated freely inside the artificial canal until the fracture occurred, which was confirmed both visually and audibly, and the time recorded on a digital chronometer.

### Statistical analysis

The normal distribution of Sa, Sq, Sz, Ssk and time to fracture data was confirmed by the Shapiro-Wilk test (p>0.05). ANOVA and Tukey tests were performed for intra-group analysis (unused conditions, after the first use and after the second use). The t-test was performed for inter-group analysis. For the cyclic fatigue test, Student t test were used for both intra and intergroup analysis. All statistical procedures were performed with a cutoff for significance at 5% using the BioEstat 5.0 software (Instituto Mamirauá, Belém, PA, Brazil).

## Results

### NiTi Instrument analysis in Light Interferometry Profiler

The data obtained by the quantitative analysis are shown in Table 1. In the analysis between the groups, there was a statistically significant difference (p<0.05) in the unused condition in the Sa and Sq parameters, where the Reciproc group showed higher values. No significant difference was observed in the other tested parameters for unused conditions or for all tested parameters after one or two retreatment procedures. The results are summarized quantitatively in Table 1 and qualitatively in Figure 01.

**Table 1:** Mean and standard deviation of the Sa, Sq, Sz and Ssk parameters of tested instruments at the different time-points.

	Unused condition	First use	Second use
Reciproc			
Sa	0.42 ± 0.05^a*^	0.46 ± 0.07^a^	0.44 ± 0.06^a^
Sq	0.55 ± 0.07^a*^	0.61 ± 0.10^a^	0.57 ± 0.08^a^
Sz	4.30 ± 0.93^a^	4.82 ± 0.91^a^	4.41 ± 0.97^a^
Ssk	-0.01 ± 0.31^a^	0.04 ± 0.26^a^	-0.05 ± 0.23^a^
Pro R			
Sa	0.33 ± 0.05^a^	0.44 ± 0.14^a^	0.32 ± 0.06^a^
Sq	0.47 ± 0.11^a^	0.60 ± 0.18^a^	0.45 ± 0.09^a^
Sz	5.13 ± 2.20^a^	4.95 ± 1.05^a^	3.87 ± 0.75^a^
Ssk	0.62 ± 1.15^a^	0.33 ± 0.26^a^	0.17 ± 0.26^a^

* Represents significant difference between the different tested instruments in the same time-point (p<0.05). Equal superscript letters represent no significant difference between the same instrument and parameter at different evaluation time-points (p>0.05)

**Figure 1:**
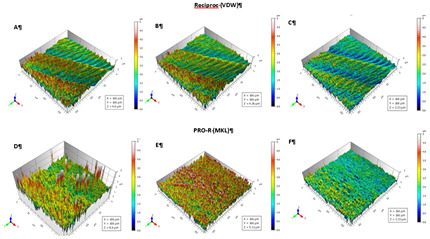
Flute surface area of a Reciproc instrument at unused condition (a), and after the 1st use (b) and after 2nd use (c) instrumentation cycles. Flute surface area of a Pro-R instrument at unused condition (d), and after the 1st use (e) and after 2nd use (f) instrumentation cycles.

### Scanning electron microscopy (SEM)

**Figure 2:**
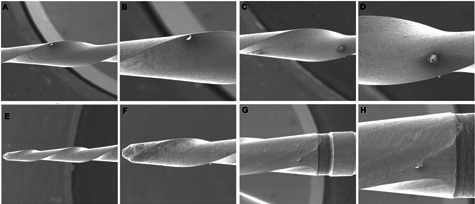
Superficial debris observed on Pro R (A, B, C e D) and Reciproc (E, F, G e H) representative samples of instruments after 2nd use. The two columns on the left and the two on the right represent magnification at x100 and x250, respectively.

Considering topographic features in both instruments, after the second use, no major change or deformation was observed on the used instruments. Few debris accumulations was observed in the samples (Figure 2).

### Cyclic fatigue

In the cyclic fatigue test, both new and used Reciproc instruments showed a longer time to fracture when compared to Pro-R instruments (p<0.05). However, while statistical differences were observed in the cyclic fatigue between new and used Pro R instruments (p<0.05), no differences were observed between new and used Reciproc instruments (p>0.05) (Table 2).

**Table 2:** Mean and standard deviation of the cyclic fatigue testing.

Instrument	New	Second use
Reciproc	182 ± 27^Aa^	169 ± 33^Aa^
ProR	136 ± 44^Ba^	98 ± 31^Bb^

Different uppercase letters in the same column represents statistical differences between the different instruments in the same test condition (p<0.05), while different lowercase letters in the same row represents statistical differences between the same instrument in different tested conditions (p<0.05).

## Discussion

The evaluation of the topographic characteristics of the surfaces of NiTi provides a better understanding of its properties and clinical performance (11, 16). Changes on the surface roughness after use can reduce the cutting efficiency of the instrument and increase the likelihood of fracture ^16^.

This study defined the analysis area 3mm from the tip of each instrument, as previously determined ^15^ ^,^ ^19^. This region is considered to be one of the most subjected to severe cyclic load conditions during root canal treatment especially in curved roots ^20^ ^,^ ^21^ and may be the area most susceptible to fractures ^22^. The evaluation of the instruments in three moments, unused, after the first use and after the second use, was also previously performed during root canal treatment procedures ^17^ ^,^ ^19^. The analysis in more than one cycle is justified, because, although the manufacturer recommends the single use of the files used in this study, usually the same instrument ends up being used in the preparation of all canals of the same tooth, as in molars with 3 or 4 root canals, and with sometimes complex anatomies ^9^ ^,^ ^17^. A recent study using Reciproc instruments showed a greater number of instruments fracture in molars when compared to uniradicular or biradicular teeth ^23^. This finding is related to the anatomic characteristic of such teeth, which is usually more complex than anterior teeth, and also due to the number of root canals. These results corroborate with the importance of evaluating NiTi instruments properties after use, to avoid instruments fracture. However, it is worth mentioning that the literature reports that mandibular canines have a mesiodistal narrow-shaped root canal, but frequently extensive buccolingually ^24^. Such complexity motivated the use of this group of teeth in this research.

In the present study, the selection and preparation of the teeth were performed in order to standardize them in the following way: teeth from the same group (mandibular canines) with similar dimensions adjusting of #15 K file assessed by tactile perception because if the instrument used for the first treatment did not significantly touch the root canal walls, the instrument used for retreatment could easily remove the filling material. In addition, the removal of the teeth crowns to homogenize the teeth length. It is worth mentioning that the decoronation procedure does not invalidate the results found, since it can simulate clinical situations of teeth with great coronary destruction ^25^. The root canal retreatments were performed on single-rooted human teeth in order to approach the clinical reality. Although other studies have chosen simulated resin root canals, stating that this method promotes greater standardization of length, curvature and diameter ^15^ ^,^ ^17^ ^,^ ^19^ ^,^ ^26^. It is well known that the dentin hardness of human teeth is different from the hardness found in simulated resin root canals ^27^ which could directly influence the wear of the instrument.

As for the evaluation method, three-dimensional noncontact light interferometer profiler allows the topographic characterization of surfaces, providing high-resolution three-dimensional images, regardless of the type of surface: flat, curved, rough or smooth. In this methodology, there is no interference from the operator due to the automatic measuring cycle of the equipment. This technique allows quick measurements, without causing damage to the studied material, it has an extended scanning range to measure profile heights from <1 nm to 20,000 μm, with high vertical optical resolution (*z*-axis) of 0.01 nm, lateral resolution (*x*- and *y*-axes) of 0.4-0.6 μm and a repeatability (Z scanning) of 0.02 nm, which allows reliable analyzes to be made at different times ^15^ ^,^ ^17^ ^,^ ^19^ ^,^ ^28^. Besides that, the definition of the rotational reference allows measurements to be made in the same surface area at different moments of analysis, increasing the accuracy of comparisons. Barbosa et al. ^29^ reported that the methodologies available for surface evaluation of endodontic instrument damage samples (Scanning Electron Microscopy - SEM and Atomic Force Microscopy - AFM), do not allow quantitative assessment (SEM) and only evaluate flat and rigid surfaces (AFM).

The results of the present study demonstrated that the Sa and Sq value of Reciproc instruments in unused condition were higher when compared to Pro-R instruments. Such results corroborate with the findings of Ferreira et al. ^17^, who found changes and irregularities on the flute surface. These changes were mainly milling marks and small pits that occurred before using Reciproc instruments. Hanan et al. ^11^, using SEM analysis, also observed the presence of superficial irregularities in the cutting blades of these instruments before use. These changes in the metallic surface before use could have resulted from industrial processes. They are considered homogeneity disorders that could impair the physical-mechanical properties of these materials and compromise their integrity during clinical use, making the file more susceptible to fracture ^30^ ^,^ ^31^. However, it is worth mentioning that Özyürek & Demiryürek ^3^ found lower rates of deformation and fracture of Reciproc instruments during root canal retreatment procedures.

Regarding the effect of use on the topographic analysis, Ferreira et al. ^17^ and Barbosa et al. ^19^ showed significant differences between the instruments evaluated without use and after two uses. This study, on the other hand, evaluated the instruments after retreatment procedures and, although it did not find any statistically significant difference in relation to the number of uses, the surface changes, in numeric values, could be observed in both the Reciproc and Pro-R systems. These irregularities could be described as deformations, steps, grooves, microcavities and debris ^15^ ^,^ ^17^ from the retreatment procedure. However, SEM analysis did not detect major differences in the Pro-R and Reciproc instruments after two uses, showing only the presence of some few amount of debris.

As complementary analysis cyclic fatigue tests comparing new and used instruments were also performed. The results of such tests demonstrated that Reciproc instruments showed higher cyclic fatigue resistance when compared to Pro-R, in both tested conditions - new and second used instruments. While no previously published study compared both systems, there are a plethora of studies demonstrating the good performance of Reciproc instruments in cyclic fatigue tests. A recent study ^32^ compared Reciproc with replica-like instruments and showed better results of the former. The better results of Reciproc can be explained by slight differences mainly on its NiTi alloy and instrument design. Interestingly, while new and used Reciproc did not show differences in the cyclic fatigue tests, Pro-R showed lower cyclic fatigue resistance after two uses, indicating the higher risk to fracture of this instrument.

These findings corroborate those of Scelza et al. ^33^, indicated that the Reciproc system obtained good results regarding static and dynamic cyclic fatigue. However, instrument fracture remains a concern. According to Tzanetakis et al. ^34^, there is a higher frequency of instrument fracture during retreatment procedures. The imperfections resulting from the manufacturing process compounded by the challenges encountered during the retreatment procedure, such as canal constrictions, previous procedural mishaps or resistance of the filling materials, would result in greater stress on the instrument ^35^.

The present study has some limitations that should be emphasized. First, we used human teeth with no information of the age of the donor. Certainly, such a condition would imply a change in the dentin characteristic, which could influence the type of wear on the instrument. Moreover, SEM was performed after being washed in an ultrasonic vat, which can justify the low amount of debris in the evaluated instruments. However, such ultrasonic bath is essential for the light interferometry profiler evaluation. In addition, due to the destructive nature of cyclic fatigue tests, it was not possible to evaluate the instruments after one use.

The use of a three-dimensional noncontact light interferometer profiler was able to accurately provide reliable data at the nanoscale. Therefore, it is suggested that the present methodology can be of great value to the evaluation of new instruments that appear every day in the scope of endodontics, leading the professional to make safer decisions in the clinic.
